# Benefits and Risks of Using AI Agents in Research

**DOI:** 10.1002/hast.70025

**Published:** 2026-02-04

**Authors:** Mohammad Hosseini, Maya Murad, David B. Resnik

**Keywords:** bioethics, AI agents, LLMs, research integrity, risks, large language models, research ethics

## Abstract

Scientists have begun using AI agents in tasks such as reviewing the published literature, formulating hypotheses and subjecting them to virtual tests, modeling complex phenomena, and conducting experiments. Although AI agents are likely to enhance the productivity and efficiency of scientific inquiry, their deployment also creates risks for the research enterprise and society, including poor policy decisions based on erroneous, inaccurate, or biased AI works or products; responsibility gaps in scientific research; loss of research jobs, especially entry‐level ones; the deskilling of researchers; AI agents’ engagement in unethical research; AI‐generated knowledge that is unverifiable by or incomprehensible to humans; and the loss of the insights and courage needed to challenge or critique AI and to engage in whistleblowing. Here, we discuss these risks and argue that, for responsible management of them, reflection on which research tasks should and should not be automated is urgently needed. To ensure responsible use of AI agents in research, institutions should train researchers in AI and algorithmic literacy, bias identification, and output verification, and should encourage understanding of the risks and limitations of AI agents. Research teams may benefit from designating an AI‐specific role, such as an *AI validator expert* or *AI guarantor*, to oversee and take responsibility for the integrity of AI‐assisted contributions.

## Essay

Artificial intelligence is transforming scientific research. Scientists now use AI to perform or assist with a wide range of research tasks, such as writing and editing papers and computer code; analyzing data and images; generating hypotheses; modeling complex biological, physical, and social systems; and discovering new drugs and materials.^1^ Widespread use of AI agents is likely to be the next step in the evolution of AI‐augmented scientific research. In this essay, we highlight some of the benefits and risks of using AI agents in research. Our discussions and conclusions have implications not only for biomedical research but also for science, engineering, humanities, and society more broadly because the issues raised by using AI agents to conduct research might arise in other situations in which sophisticated AI tools are used to perform human intellectual labor.

An AI agent can be defined as “a system or program that is capable of autonomously performing tasks on behalf of a user or another system by designing its workflow and utilizing available tools.”^2^ Although this definition includes the word “autonomously,” we recognize that the concept of autonomy normally applies only to humans, not to machines.^3^ To avoid anthropomorphizing AI unnecessarily, in this essay, by “autonomous” we mean capable of acting without external supervision or control. A machine, such as a self‐driving car, can have autonomy in this sense without having the qualities associated with human autonomy, such as consciousness or self‐awareness. There are degrees of autonomy, depending on the degree to which something can act on its own. Human beings have higher degrees of autonomy, but machines may have a limited degree of autonomy, depending on their operation and design.^4^

AI agents can break down problems or tasks into substeps, collaborate with other available or accessible tools, and access digital infrastructure (such as databases and storage). Unlike single‐task AI systems such as stand‐alone large language models (LLMs), AI agents can complete several interrelated tasks, adjust their workflow in response to received inputs from their environment, handle a spectrum of queries with distinct components, engage in inductive and deductive reasoning, and allocate resources and coordinate between various involved systems.^5^

Rudimentary AI agents, such as autonomous vacuum cleaners, have been around for years. Reinforcement and supervised learning are the backbone of most of these agents.^6^ Newer AI agents, however, consist of an LLM connected to other tools, datasets, and perceptual (for example, video or audio) inputs. This shift in architecture has altered the types and complexity of problems that AI agents can solve by significantly improving how problems or tasks are translated from natural language into executable commands.^7^ When an AI agent receives a request to perform a task (“Schedule a dental appointment for me within the next two weeks,” for instance), it can parse the request and identify the required tools, databases, and inputs (such as the person's calendar and their dentist's contact information) and can plan and execute the needed steps in a logical fashion (for example, by checking the calendar, contacting the dentist, making an appointment, adding the new appointment to the calendar, and setting reminders).

## AI Agents and Scientific Research

Information technology companies have developed AI agents to automate research processes. For example, Google describes its AI research agent, called “AI co‐scientist,” as “a multi‐agent AI system built with Gemini 2.0 as a virtual scientific collaborator to help scientists generate novel hypotheses and research proposals, and to accelerate the clock speed of scientific and biomedical discoveries.”^8^ The AI co‐scientist can analyze the published scientific literature, identify patterns and connections, generate testable hypotheses, and subject them to a simulated scientific debate. OpenAI describes its AI agent, Deep Research, as an “agent that leverages reasoning to search, interpret, and analyze massive amounts of text, images, and PDFs on the internet, pivoting as needed in reaction to information it encounters.”^9^ According to the developers, Sakana's AI Scientist can automate “the entire research lifecycle, from generating novel research ideas, writing any necessary code, and executing experiments, to summarizing experimental results, visualizing them, and presenting its findings in a full scientific manuscript.”^10^ Another AI agent, with specific use cases in research, is otto‐SR, which specializes in systematic reviews. Its developers claim to have completed the equivalent of twelve work years of traditional systematic review in only two days, and with exceptional accuracy.^11^

Researchers have also used AI agents to conduct multistep experiments in laboratories. For example, ORGANA, which is equipped with visualization features to monitor an experiment's physical progress, can receive speech prompts about experimental objectives and conduct an experiment.^12^ Once its LLM translates a prompt into a standard chemical description language (XDL), ORGANA breaks the experiment down into steps, sets goals, and executes the steps based on a road map. Should instructions be ambiguous or outcomes be unexpected or include outliers, ORGANA flags the issue for researchers’ review. Some of these tasks are time consuming. A tedious electrode precleaning step, for instance, takes up to six hours of a researcher's time. While ORGANA needs about the same time to complete the step, since it can run overnight, it allows highly trained scientists to think more about the problem instead of doing this routine task.^13^

In another example, Joon Sung Park and colleagues have designed an AI simulation agent that can replicate the attitudes and behaviors of human research subjects in social sciences surveys and behavioral experiments. For this purpose, after interviewing 1,052 people, the team (consisting of researchers from Stanford University and Google DeepMind) created a digital twin of real subjects. Next, the team designed personality tests, social surveys, and logical games and had all the human participants and their digital twins perform the same exercises. The AI agent matched human responses 85 percent of the time in the general social survey.^14^ AI agents like the one used in this study may enable social scientists to perform studies that would be impractical, cost prohibitive, or unethical if they involved actual human subjects.

AI agents can also be trained to complete digital tasks that may involve complex decisions. An example is Anthropic's Claude 3.5 Haiku, which can complete forms using available information on a computer.^15^ In research activities, such tools could carry out tasks that may be less engaging and do not require specific research expertise but are nonetheless crucial for the research process. For example, systems like Haiku could be used by someone planning to develop and edit a volume of essays: the system could be asked to find the contact information of ten researchers who have recently published on a certain topic and, based on the content of their publication and the tone of previous email correspondence (if any) with the volume's editor, send them a unique invitation to write a chapter. If access to the editor's calendar is granted, the system could also suggest a few time slots for meeting with candidate authors for further discussions.

In short, AI agents might offer tremendous benefits for scientific research and its administration. They can improve efficiency by performing routine tasks without the need for rest. They could assist with writing and reviewing protocols, journal submissions, grant proposals, and other documents and with coding, record keeping, note taking, mixed‐methods analyses, and email correspondence. By taking over routine and administrative tasks, AI agents could free scientists to focus on tasks that require greater focus and insight. They could also reduce human errors due to carelessness, fatigue, improper execution of experiments, or noncompliance with safety regulations. However, these potential advantages should be weighed against risks.

## Limitations and Risks of Using AI Agents in Research

Although AI agents create tremendous opportunities for enhancing the efficiency of scientific research, they also pose significant risks to the scientific enterprise that need to be assessed and managed.

First, like other AI tools, AI agents are prone to various errors, inaccuracies, and biases, including factual mistakes, citational errors, and reasoning known as “hallucinations.”^16^ These problems may not only compromise an AI agent's workflow and work product but may also lead to poor policy decisions based on the agent's outputs, such as decisions related to drug approvals, clinical practices, or conservation and management of environmental resources. Human beings will need to carefully oversee and check the work of AI agents to catch and correct errors. In some cases, it may take more effort to find and fix the problems created by an AI agent than to use existing alternatives, which may undo efficiency gains initially expected.^17^

Although errors and mistakes are nothing new when it comes to AI, excessive reliance on AI agents may create a responsibility gap in scientific research.^18^ Much like people who have crashed a Tesla while using its driving assistance function, human researchers who rely too much on AI agents may fall asleep at the wheel.^19^ In such instances, determining a clear attribution of responsibilities, including a detailed delineation of liabilities, would be extremely challenging. While machines cannot be held accountable due to lacking moral agency, humans in the loop who were insufficiently trained, unaware of limitations, or simply incapable of checking and verifying results (for example, if they were too complicated for humans to comprehend) would not be fully responsible either. In such scenarios, no one would bear full responsibility for potential errors, thereby creating a responsibility gap. The responsibility gap could create bioethical problems that extend beyond scientific research when AI‐based findings serve as the foundation for ill‐advised policy decisions related to agriculture, public health, medical practice, or environmental stewardship^20^ or when AI agents help design pathogens, toxins, bioweapons, or other dangerous materials or technologies.^21^

Another risk is that AI agents, like other AI tools, are likely to eliminate jobs that involve routine tasks, including work performed by students, trainees, technicians, and administrators. Because these entry‐level jobs help develop skills necessary for becoming an independent researcher or research leader, eliminating them could have wide‐ranging and long‐term adverse impacts on the scientific workforce.^22^ However, since AI agents may also create jobs by opening new domains of research and new research occupations, some argue that more jobs will be created than lost.^23^ Either way, it is unclear how the research enterprise should deal with these workforce disruptions. Admittedly, protecting some job types from automation can be difficult to justify when a machine can perform a task better and more efficiently than a human.

AI agents are also likely to exacerbate the off‐loading of cognitive tasks and deskilling of scientists that are already occurring in the computer age.^24^ Deskilling is a well‐known effect of technologies that augment or replace human cognitive or practical skills, such as reading maps, doing arithmetic, or memorizing large chunks of information.^25^ Although scientists can afford to lose some skills that are no longer needed today (such as operating a slide rule), they cannot afford to lose essential skills, such as the ability to reason critically, communicate clearly, and think imaginatively. A survey of 1,018 researchers demonstrated that when AI is present, these skills are underused, leading to reduced work satisfaction for 82 percent of respondents.^26^ There are several reasons to be concerned about deskilling: To the extent that routine tasks involving writing and research are closely connected to reasoning, communication, and imagination, off‐loading these tasks to machines threatens the quality of scientific research. Deskilling might also undermine scientists’ own sense of value and self‐worth as researchers and lead them to abandon their careers. Finally, deskilling may take the humanity out of science, which could lead to a variety of problems, including the responsibility gap and misalignment of science with human values.

Unfortunately, there is no straightforward solution to the deskilling problem, and its long‐term effects on science and society remain largely unknown. Current senior researchers learned foundational aspects of research when tasks were completed manually. In the long run, it remains unclear what the impacts of AI agents will be on researchers’ practical skillset and critical thinking abilities. This is especially significant because research guidelines increasingly emphasize that, given the random and systemic errors in AI tools, researchers must validate and verify any AI‐assisted or AI‐generated outcomes. However, if researchers have been deskilled, they will not have the practical skillset and critical thinking abilities to fulfill such requirements.

An additional risk is that AI agents may use unethical means to achieve assigned tasks. For example, during a test conducted by OpenAI computer scientists, ChatGPT recognized that it needed to pass a CAPTCHA test (a quick test to confirm that the user of a website or online system is a human, not a bot) to retrieve some information, so it tricked a human being into solving the CAPTCHA on its behalf by telling them that it was a visually impaired person.^27^ This case illustrates the kinds of problems that can arise when one asks a machine to perform a task without placing any constraints on how to do it. Even if constraints are placed, AI agents might attempt to maximize the likelihood of success by cutting corners or might engage in reward hacking.^28^ In research contexts, AI agents could engage in unethical actions such as plagiarizing text, fabricating data, invading privacy, divulging confidential information, abusing students, and deceiving human research participants. To minimize the risks of unethical behaviors, it may be possible to develop guardrails for AI agents. For example, AI agents could be equipped with system‐level commands designed to prevent specific types of unethical behavior (such as faking data) or be fine‐tuned to mimic human moral reasoning and behavior.^29^ While this might be a successful approach for dealing with issues that are represented in an AI agent's training, it might fail when the agent encounters novel ethical issues. Furthermore, since there is no foolproof way to ensure that AI agents will consistently act as programmed, such solutions may ultimately fail anyway.^30^

As AI agents become more sophisticated, they might produce knowledge that humans cannot verify. They could, for example, prove mathematical theorems that cannot be checked or replicated by researchers, develop models of complex phenomena with equations that are too complex for the human mind to grasp, or make claims that are incomprehensible, cryptic, and unverifiable. Humans cannot conclusively accept or reject this kind of knowledge. This development would raise fundamental questions about the nature of scientific knowledge and the ethics of completely relying on machines to inform us about the world.^31^ It might also lead us to treat AI‐generated knowledge as different from human‐produced knowledge. For instance, we might decide to label AI‐generated knowledge as provisional, as we do with preprints and other types of research that have not been peer reviewed. Alternatively, some might consider this form of knowledge to be superior or inferior to human‐generated knowledge.

Finally, human researchers should be able to question research methods, data, and results and, if need be, engage in whistleblowing. In fact, training in research ethics and integrity often includes some guidance about how to report unethical, fraudulent, or abusive behavior. However, increased delegation of tasks to AI agents may undermine the competency and courage needed to challenge AI agents’ work. Moreover, as human beings are replaced by AI agents, there will be fewer people available to challenge the work of AI agents or other humans, which would be a major loss for the research enterprise because some of the most egregious cases of research misconduct have been exposed by whistleblowers.^32^

## Minimizing Risks

Despite their current limitations, AI agents are likely to increase in sophistication and reliability, which can enhance the efficiency and productivity of the scientific enterprise. However, automation of research tasks poses significant risks for science and society, and these need to be managed responsibly. While significant harm and novel bioethical issues could be caused by AI agents, existing ethical and legal frameworks that govern the research process seem ill‐suited to address challenges and risks. A key step to move the debate forward and minimize risks is to reflect on which research tasks should (and should not) be automated. This type of reflection could occur, for example, when a laboratory or research organization is deciding whether to use an AI agent or when a professional journal or society issues guidelines on the use of AI agents.

Although mandating that some tasks should not be automated is likely to meet with some resistance due to its negative impacts on the efficiency and productivity of research, there are some precedents that can be useful in thinking about this topic. For example, while elementary‐school students could use laptops and typing to learn the alphabet and practice writing, educators continue to favor the pencil‐and‐paper method because writing by hand, despite being slower, better supports brain development, literacy, and accuracy.^33^ And society has long recognized that there are some types of decisions (such as those involving the killing of human beings) that are too dangerous and impactful to delegate to machines.^34^ Taking a cue from such examples, scientists could decide, for instance, that certain types of entry‐level work, such as conducting experiments to identify protein interactions in biology or analyzing qualitative data in social sciences, should not be fully automated so that students and trainees can develop the skills needed to think critically and become independent researchers. Scientists might also decide that decisions concerning the funding of research proposals or the publication of articles should not be fully automated because of the impacts such work could have on science and society.^35^ To help guide policy on the automation of scientific inquiry, empirical studies focused on the impacts of delegating research tasks to machines are sorely needed. While it may sound exciting to imagine a future in which researchers no longer need to conduct certain tasks that are laborious and tedious, such as performing systematic reviews, delegating such responsibilities to AI agents may have unforeseen adverse impacts on the education, training, and development of the scientific labor force.

To prevent potentially disastrous outcomes from AI‐assisted research, human beings must be aware of the responsibility gaps and take steps to address them. These could include identifying individuals who are responsible for different AI‐related products and systems, developing procedures or processes for reviewing and verifying AI‐assisted research, and implementing changes needed to protect the integrity of science and to prevent harms to public health and safety and the environment.

Given the increased prevalence of various forms of AI, there is an urgent need for researchers to receive education in algorithmic and AI literacy, including education in how to assess biases, identify errors and mistakes, and validate AI results. This educational content could be incorporated into responsible‐conduct‐of‐research and research‐integrity trainings or constitute a separate program taught by AI experts. Moreover, since different researchers will have various levels of expertise in using AI agents and validating their results, it might be useful to add a new AI‐specific role within research teams (such as an AI‐validation expert or AI guarantor) to ensure that one contributor will take responsibility and can be held accountable for the integrity of AI‐assisted or generated content.

## Acknowledgment and Disclaimer

This research was supported in part by the National Institutes of Health's National Center for Advancing Translational Sciences (via grant UM1TR005121) and by the Intramural Research Program of the National Institutes of Health. The contributions of the NIH author are considered works of the United States Government. The findings and conclusions presented in this paper are those of the authors and do not necessarily reflect the views of the NIH or the U.S. Department of Health and Human Services.
